# Global research progress of visceral hypersensitivity and irritable bowel syndrome: bibliometrics and visualized analysis

**DOI:** 10.3389/fphar.2023.1175057

**Published:** 2023-05-02

**Authors:** Siyu Tian, Hang Zhang, Siqi Chen, Pengning Wu, Min Chen

**Affiliations:** ^1^ School of Clinical Medicine, Chengdu University of TCM, Chengdu, China; ^2^ Department of Colorectal Diseases, Hospital of Chengdu University of Traditional Chinese Medicine, Chengdu, China

**Keywords:** IBS, visceral hypersensitivity, bibliometrics, visualized analysis, research progress

## Abstract

**Background:** Irritable bowel syndrome (IBS) is a group of functional intestinal disorders characterized by abdominal pain, bloating, and changes in bowel habits, and/or stool characteristics. Recent studies have shown that there has been a significant advancement in the study of visceral hypersensitivity in IBS. Through the use of bibliometrics, this study aims to provide a comprehensive overview of the knowledge structure and research hotpots of visceral hypersensitivity in IBS.

**Methods:** Publications related to visceral hypersensitivity in IBS from 2012 to 2022 were searched on the web of science core collection (WoSCC) database. CiteSpace.6.1. R2 and Vosviewer 1.6.17 were used to perform bibliometric analysis.

**Results:** A total of 974 articles led by China and the United States from 52 countries were included. Over the past decade, the number of articles on visceral hypersensitivity and IBS has steadily increased year by year. China, the United States, and Belgium are the main countries in this field. Univ Oklahoma, Univ Gothenburg, and Zhejiang University are the main research institutions. Simren, Magnus, Greenwood-van meerveld, Beverley, and Tack, Jan are the most published authors in this research field. The research on the causes, genes, and pathways involved in visceral hypersensitivity in IBS and the mechanism of IBS are the main topics and hotspots in this field. This study also found that gut microbiota may be related to the occurrence of visceral hypersensitivity, and probiotics may be a new method for the treatment of visceral hypersensitivity and pain, which may become a new direction for research in this field.

**Conclusion:** This is the first bibliometric study to comprehensively summarize the research trends and developments of visceral hypersensitivity in IBS. This information provides the research frontier and hot topics in this field in recent years, which will provide a reference for scholars studying this field.

## 1 Introduction

Irritable Bowel Syndrome (IBS) is a functional gastrointestinal disorder which the main clinical symptoms are abdominal pain, bloating, and changes in bowel habits and/or fecal characteristics (Ford et al., 2020). IBS is generally believed to affect 2%–15% of people in Western or Asian nations (Choung & Locke, 2010). The occurrence of IBS causes a huge burden on patients’ life and the economy of society (Zhou & Verne, 2011). According to statistics, there are 3.1 million outpatient consultations for IBS each year in the US, costing a total of more than $20 billion (Chey et al., 2015). The annual direct and indirect costs associated with IBS are estimated to be as high as 8 billion euros in Europe (Flacco et al., 2019) and as high as 123 billion yuan in China (Zhang et al., 2016). The first-line therapy for IBS includes dietary changes, soluble fiber, and antispasmodic medications (Ford et al., 2018). For patients with severe symptoms, treatment includes central neuromodulators, including low-dose tricyclic antidepressants, gut secretokines, opioid or serotonin-receptor medications, antibiotics, and psychotherapy (Ford et al., 2018).

IBS’s pathophysiological mechanism is poorly known. Possible causes include immunological variables, gut microbiota alterations, genetic factors, visceral hypersensitivity, gut-brain axis, and psychosocial comorbidities (Sultan and Malhotra, 2017). Available evidence suggests that a portion of IBS patients (between 30 and 40 percent) have a higher sensitivity to colon dilatation, which is manifested by a lower pain threshold and a rise in sensory intensity in response to colon dilation (Mayer & Gebhart, 1994; Bouin et al., 2001). Hence, there is a link between the mechanism of IBS and visceral hypersensitivity, which can be served as a clinical marker of IBS and explain the symptoms of constipation, bloating, and abdominal discomfort experienced by these individuals (Zhou & Verne, 2011). Visceral hypersensitivity includes hyperalgesia and allodynia, and the pathogenesis of visceral hypersensitivity is not fully understood (Barbara et al., 2011). Sensitization of peripheral and central afferent nerve pathways, minor inflammatory responses, psychosocial variables, and modifications in gut motor function is now the primary pathogenesis of visceral hypersensitivity (Akbar et al., 2009). Current studies have found that various mechanisms may be involved in the occurrence of visceral hypersensitivity. Immune cells in the intestinal mucosal wall, such as mast cells, can hypersensitize afferent nerves by releasing their mediators, leading to the occurrence of pain and other symptoms (Azpiroz et al., 2007). In addition, increased intestinal mucosal permeability, changes in gut microbiota, and dietary habits may also contribute to the development of visceral hypersensitivity (Deiteren et al., 2016).

With the development of gastroenterology, the relationship between IBS and visceral hypersensitivity has attracted more and more attention. However, bibliometrics and visual analysis have not been used to understand the research trends and hotspots in this field. Bibliometric analysis refers to the quantitative analysis of all knowledge in a subject using mathematical and statistical knowledge (Ma et al., 2021). Bibliometric analysis plays an important role in reflecting characteristics and future trends (Zou and Sun, 2021). This study aims to comprehensively and systematically review the current research status of visceral hypersensitivity and IBS from 2012 to 2022 and to make up for the deficiency of bibliometric analysis in this field.

## 2 Methods

### 2.1 Data source

**FIGURE 1:**
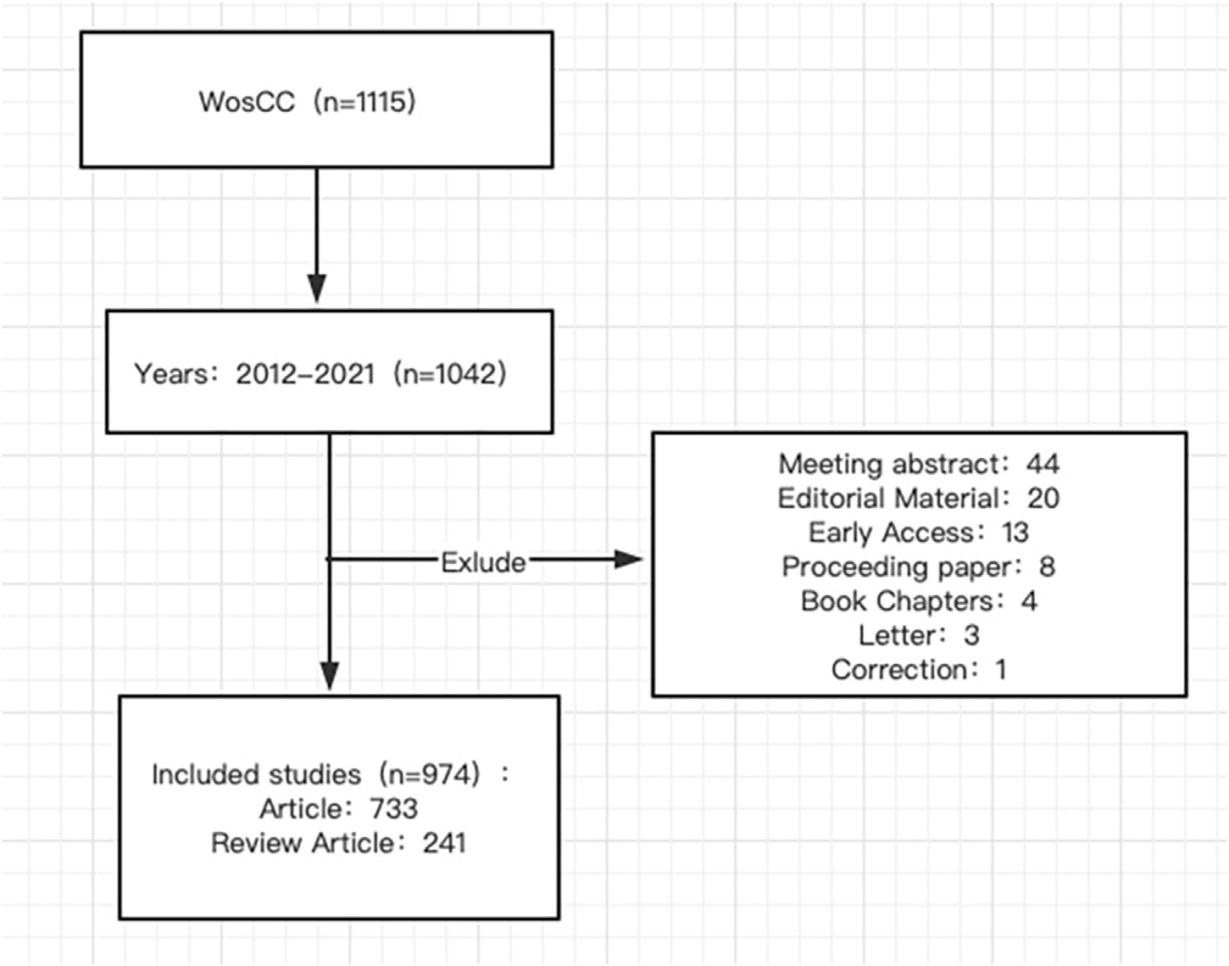
Flow diagram of the included articles.

Search for the data in the Web of Science Core Collection (WoSCC), which is regarded as one of the most widely used and comprehensive databases for citation analysis (Kulkarni et al., 2009). The search strategy is as follows. The search period was limited to 1 January 2012, to 31 December 2022. The type of literature was set as “Article or Review”. The search terms for IBS and visceral hypersensitivity were combined with subject words and free words: TS = (“visceral hypersensitivity” OR “visceral hypersensitivities” OR “visceral allergies” OR “visceral allergic reaction” OR “visceral allergic reactions”) AND TS = (IBS OR “irritable bowel syndrome” OR “irritable bowel” OR “irritable colon”). The final step is to export the retrieved literature as “complete records with cited references” and store it as a text file for further research. Two researchers worked independently to search the data on 20 February 2023. Comprehensive search results are shown in Figure 1.

### 2.2 Data analysis

For data analysis, CiteSpace.6.1.R2, Vosviewer1.6.17, and Microsoft Office Excel 2010 are utilized. Microsoft Office Excel 2010 can be used for data administration, annual publication trend statistics, and creating pertinent tables. Moreover, CiteSpace.6.1. R2 was used to draw a visual map to analyze the annual number of publications, the number of publications and the centrality of countries, institutions, and authors, the frequency and centrality of keywords, the frequency of highly cited articles, and the clusters and burst terms of keywords. Vosviewer1.6.17 is being used to analyze highly co-cited references. CiteSpace’s specific parameter settings and result explanations have been previously discussed and published (Zou & Sun, 2021). The time slice is set from the first day of 2012 to the last day of 2022. The node can represent a Country, Institution, or Author.

## 3 Results

### 3.1 Annual publications and trend analysis

**FIGURE 2:**
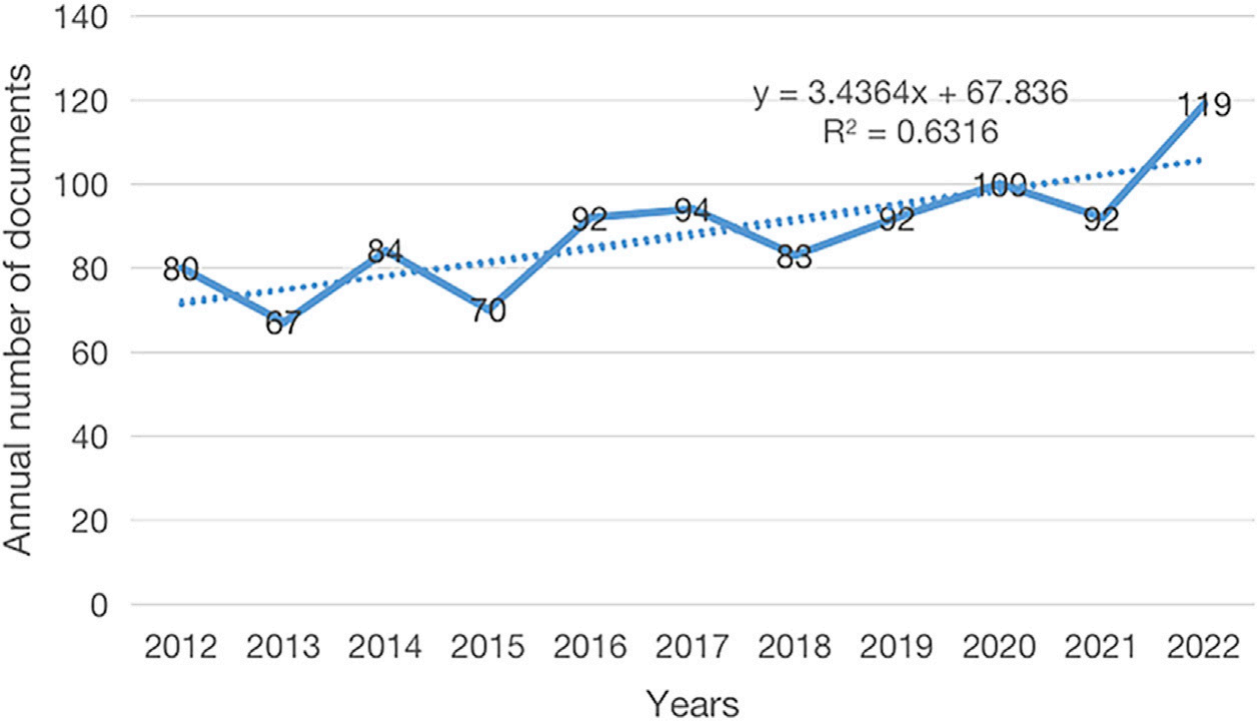
Published trend chart concerning IBS and the visceral hypersensitivity.

Between 2012 and 2022, a total of 974 articles were published, including 734 articles and 240 reviews. The number of papers published each year can indicate trends in a specific field of study. The analysis results show that the number of related papers published each year is large and growing slowly, with a peak in 2022 (*n* = 119) (Figure 2). It demonstrates that researchers are paying increasing attention to the field of visceral hypersensitivity. Furthermore, the growth trend model [Coefficient of determination (R^2^) = 0.6316] reveals a significant relationship between publication year and quantity (Figure 2). The model also forecasts there will be 109 related publications by 2023.

### 3.2 Analysis of publication trends

**FIGURE 3:**
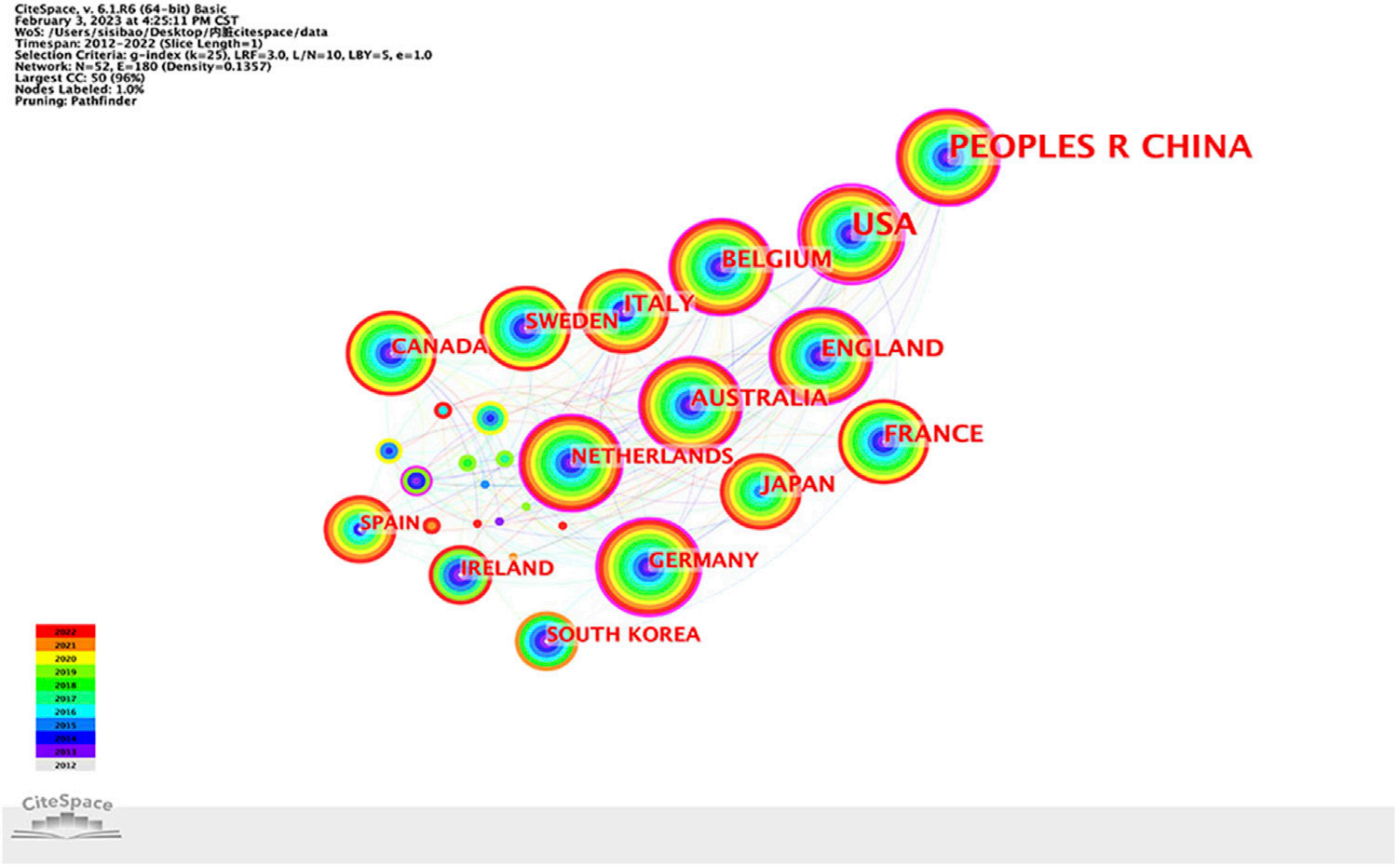
Country/region collaboration network of research on IBS and the visceral hypersensitivity.

A total of 52 countries/regions have published articles in this research field. Cooperation between countries is represented on the network map by 52 nodes and 180 links, as shown in Figure 3. More articles are published when nodes are larger. If the centrality of a country or region is greater than 0.1, there will be a purple circle outside the corresponding node on the network map. Table 1 lists the top 10 countries/regions in terms of research publications and the centrality of their publications. China had the largest number of publications (329 articles, 31.97%), followed by the United States (279 articles, 27.11%) and Belgium (67 articles, 6.51%), all of which were the key countries for the study of IBS and visceral hypersensitivity. The greater the centrality, the stronger the cooperation between countries. What we can see is that the United States (0.53), Germany (0.25), Australia (0.2), England (0.19), and China (0.18) are the five most centralized nations.

**TABLE 1:** Countries/regions, institutions, and authors ranked by publications and centrality.

Item	Rank	Name	Publications	Name	Centrality
Countries/Regions	1	China	329 (31.97%)	USA	0.53
	2	USA	279 (27.11%)	Germany	0.25
	3	Begium	67 (6.51%)	Australia	0.2
	4	England	65 (6.32%)	England	0.19
	5	France	61 (5.93%)	China	0.18
	6	Italy	56 (5.44%)	Begium	0.14
	7	Australia	51 (4.96%)	Netherlands	0.14
	8	Japan	46 (4.47%)	Switzerland	0.14
	9	Sweden	39 (3.79%)	Italy	0.08
	10	Netherlands	36 (3.50%)	Canada	0.07
Institutions	1	Univ Oklahoma	29 (14.29%)	China Acad Chinese Med Sci	0.15
	2	Univ Gothenburg	24 (11.82%)	Univ Hosp Leuven	0.14
	3	Zhejiang Univ	22 (10.84%)	Katholieke Univ Leuven	0.11
	4	Univ Calif Los Angeles	20 (9.85%)	Univ Michigan	0.1
	5	Mayo Clin	19 (9.36%)	Univ Calgary	0.1
	6	Shanghai Univ Tradit Chinese Med	19 (9.36%)	Huazhong Univ Sci and Technol	0.09
	7	Natl Univ Ireland Univ Coll Cork	19 (9.36%)	Univ Calif Los Angeles	0.08
	8	Asahikawa Med Univ	18 (8.87%)	Univ Bologna	0.08
	9	Univ Leuven	17 (8.37%)	Maastricht Univ	0.07
	10	Katholieke Univ Leuven	16 (7.88%)	Univ N Carolina	0.07
Authors	1	Simren, Magnus	17 (22.67%)	Simren, Magnus	0.01
	2	Greenwood-van meerveld, Beverley	16 (21.33%)	De man, Joris G	0.01
	3	Tack, Jan	15 (20.00%)	Tack, Jan	0
	4	Nozu, Tsukasa	14 (18.67%)	Castro, Joel	0
	5	Okumura, Toshikatsu	13 (17.33%)	Harrington, Andrea M	0

**FIGURE 4:**
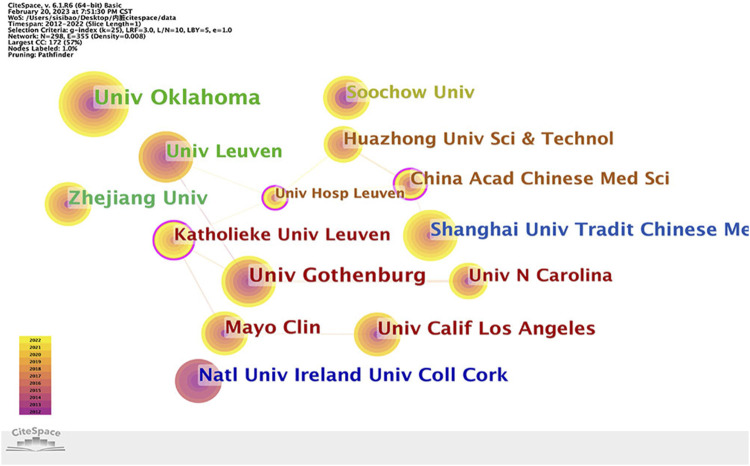
Institutions’ collaboration network of research on IBS and the visceral hypersensitivity.

298 institutions contribute to the research field. Figure 4 shows the cooperative network of institutions, which includes 298 nodes and 355 connections. Table 1 shows the top five organizations with the most publications are Univ Oklahoma (29, 14.29%), Univ Gothenburg (24, 11.82%), Zhejiang Univ (22, 10.84%), Univ Calif Los Angeles (20, 9.85%), Mayo Clin (19, 9.36%), while China Acad Chinese Med Sci (0.15), Univ Hosp Leuven (0.14), Katholieke Univ Leuven (0.11), Univ Michigan (0.1), Univ Calgary (0.1) are the top five institutions presenting the strongest centrality.

**FIGURE 5:**
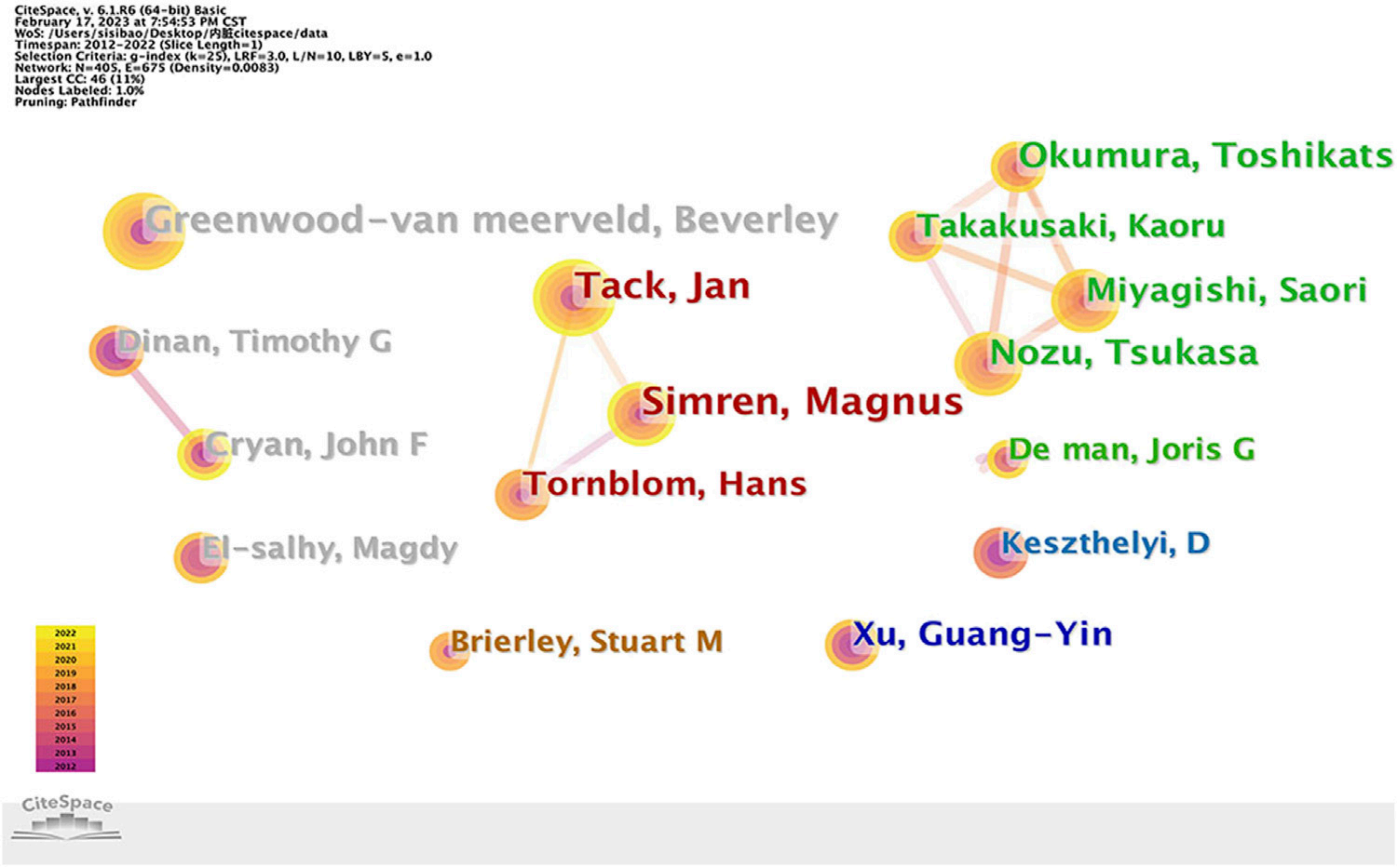
Authors’ collaboration network of research on IBS and the visceral hypersensitivity.

There are a total of 405 authors who contributed to the publications in regard to IBS and visceral hypersensitivity. In accordance with Figure 5, 405 nodes and 675 links are shown on the authors’ network map. Table 1 lists the top 5 authors who published the most articles. Simren, Magnus made the greatest contribution to the number of articles (17 publications, 22.67%), with a centrality of 0.1, followed by Greenwood-van meerveld, Beverley (16 publications, 21.33%), Tack, Jan (15 publications, 20.00%), Nozu, Tsukasa (14 publications 18.67%), Okumura and Toshikatsu (13 publications, 17.33%). Simren, Magnus, Greenwood-van meerveld, Beverley, Tack, Jan, Nozu, Tsukasa, Okumura, and Toshikatsu have made important achievements and great influence in the research field of IBS and visceral hypersensitivity.

## 4 Research topic analysis

### 4.1 Analysis of co-cited references

**FIGURE 6:**
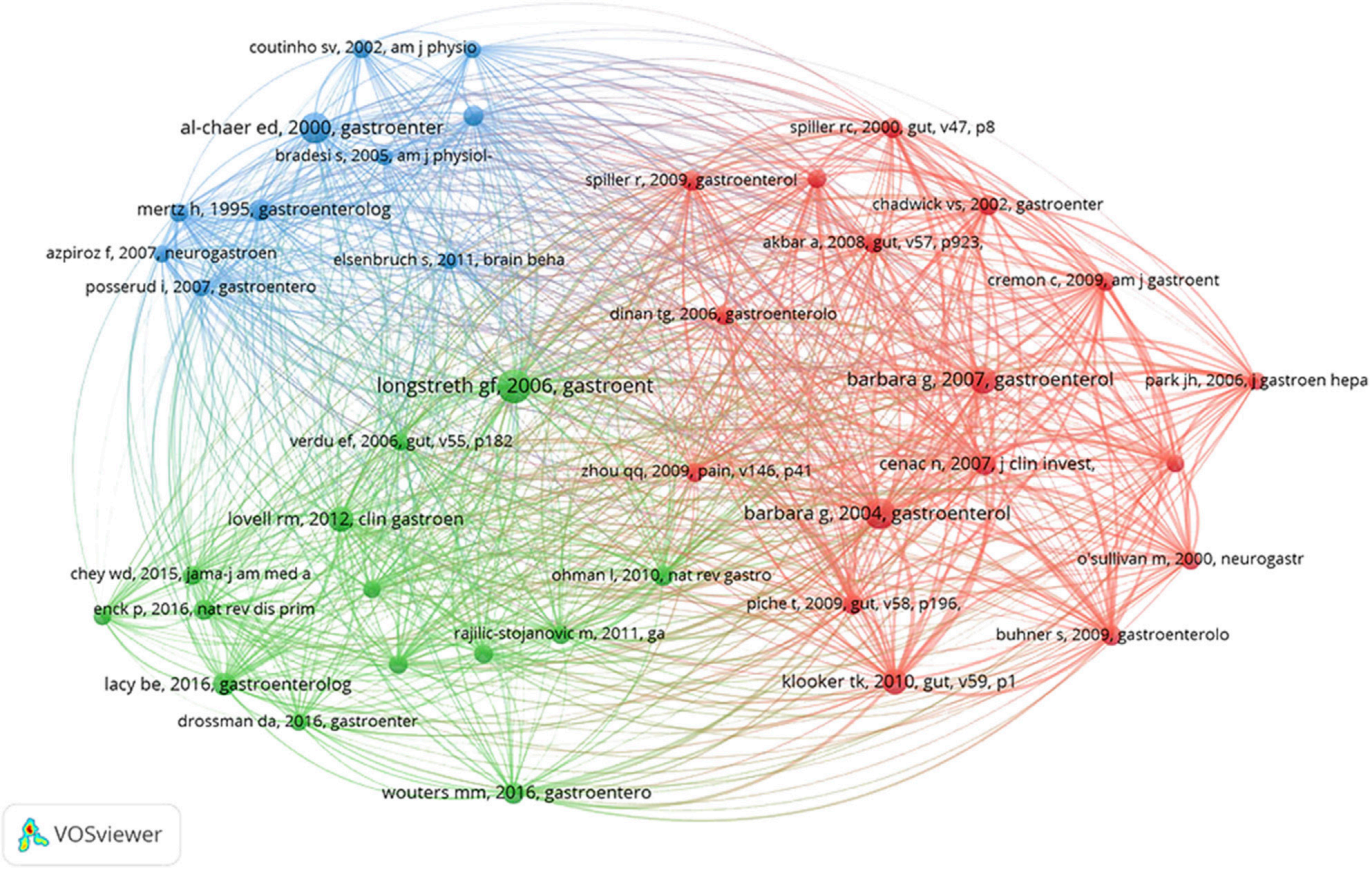
Visualization of a clustering map of co-cited references.

References that researchers cite collectively are known as co-cited references. By analysis of article citations, VOSviewer visualizes the references in the form of co-cited reference maps that highlight areas of research that IBS and visceral hypersensitivity have in common. The study cited 34871 co-cited references, according to VOSviewer’s results. When the number of citations for a cited reference was reduced to 50, 41 references remained. As shown in Figure 6, the literature is divided into three clusters in the network diagram of highly co-cited references, which correspond to three colors in the network diagram. The red cluster is mainly related to the pathogenesis of IBS, visceral hypersensitivity, and visceral pain, including activation and dependent excitation of mast cells near the colic nerve in relation to visceral hypersensitivity in IBS (Barbara et al., 2004; Barbara et al., 2007; Grabauskas et al., 2020), the pathophysiological process of visceral pain in IBS mediated by protease activity (Ceuleers et al., 2016; Zhao et al., 2022) and intestinal mucosal soluble medium (Cenac et al., 2007; Piche et al., 2009), plasma cytokines may serve as potential biomarkers for gut-brain axis disorders in IBS (Dinan et al., 2006), and TRPV1’s role in visceral hypersensitivity in IBS (Wouters et al., 2016). The literature in the green cluster is more inclined to the review of IBS and the research progress of IBS and gut microbiota. The majority of the literature in the blue cluster is concerned with basic experiments related to IBS and visceral hypersensitivity. The top 10 most frequently co-cited literature are listed in Table 2. “Functional Bowel Disorders” is the most widely co-cited paper published in Gastroenterology in 2006 (Longstreth et al., 2006), where Longstreth et al. revised the Rome II diagnostic criteria for functional bowel disease and updated recommendations for diagnosis and treatment, and it also suggested that functional and structural changes of visceral organs may lead to the occurrence of functional bowel disease. In addition, it can be found from the ten literature with the highest number of co-citations that basic research focuses on colon stimulation, mast cells of visceral pain-sensing neurons, changes in rectal perception, protease activity, and TRPV1.

**TABLE 2:** Top 10 highly co-cited references.

Item	Ranking	Title	Citation	Year
Co-reference	1	Functional Bowel Disorders	196	2006
	2	Activated Mast Cells in Proximity to Colonic Nerves Correlate With Abdominal Pain in IBS	167	2004
	3	A New Model of Chronic visceral hypersensitivity in Adult Rats Induced by Colon Irritation During Postnatal Development	162	2000
	4	Mast Cell-Dependent Excitation of Visceral-Nociceptive Sensory Neurons in IBS	121	2007
	5	Global Prevalence of and Risk Factors for IBS:A Meta-analysis	116	2012
	6	The mast cell stabiliser ketotifen decreases visceral hypersensitivity and improves intestinal symptoms in patients with IBS	111	2010
	7	Bowel Disorders	89	2016
	8	Role for protease activity in visceral pain in IBS	84	2007
	9	Altered Rectal Perception Is a Biological Marker of Patients With IBS	76	1995
	10	Histamine Receptor H1-mediated Sensitization of TRPV1 Mediates visceral hypersensitivity and Symptoms in Patients With IBS	76	2015

From the analysis of the top ten co-cited literature, it can be found that basic research focuses on the mechanism of IBS and visceral hypersensitivity, mainly including mast cells, protease activity, and TRPV1. Among the top ten pieces of co-cited articles, Giovanni Barbara’s article found that in IBS patients, an increased number of mast cells, an increased area of the intestinal mucosa occupied by mast cells, an increased tryptase, and the release of tryptase and histamine from mast cells were significantly associated with the severity and frequency of abdominal pain/discomfort (Barbara et al., 2004). Nicolas Cenac’s study found that the release of arginine site protein hydrolysate in colon biopsy tissues of IBS patients was significantly increased, and found that this proteolytic activity activated sensory neurons through PAR2-activated mechanisms, resulting in somatic hyperalgesia and visceral hypersensitivity (Cenac et al., 2007). The study by Mira M Wouters found an enhanced TRPV1 response in submucosal neurons of IBS patients. HRH1 can lead to enhanced TRPV1 response in mouse intestinal submucosal neurons. Ebastine can reduce visceral hypersensitivity and increase symptom relief (Wouters et al., 2016).

### 4.3 Analysis of high-cited references

The top 10 highly cited studies on IBS and visceral hypersensitivity were shown in Table 3. The most cited article titled “Bowel Disorders” (Mearin et al., 2016) updated the latest knowledge on the epidemiology, etiology, pathophysiology, diagnosis, and treatment of functional bowel disorders (FBD) and changed the Rome III FBD criteria, which were last published in 2006. The frequency of changes in abdominal pain is essential to the diagnosis and definition of IBS, and “Bowel Disorders” (Mearin et al., 2016) also state that immunological response and chronic stress response are two of the elements that might initiate or exacerbate IBS. In addition to the review literature on IBS, the mechanism between TRPV1 and visceral hypersensitivity, the mechanism between mast cells and visceral hypersensitivity, and the relationship between gut microbiota and visceral hypersensitivity were also in the top 10 piece of literatures.

**TABLE 3:** Top 10 highly cited references.

Item	Ranking	Title	Citation	Year
high-cited References	1	Bowel Disorders	67	2016
	2	Histamine Receptor H1-Mediated Sensitization of TRPV1 Mediates Visceral Hypersensitivity and Symptoms in Patients With Irritable Bowel Syndrome	60	2016
	3	Irritable bowel syndrome	56	2016
	4	Global prevalence of and risk factors for irritable bowel syndrome: a meta-analysis	45	2012
	5	Irritable bowel syndrome: a clinical review	44	2015
	6	Functional Gastrointestinal Disorders: History, Pathophysiology, Clinical Features and Rome IV	42	2016
	7	The mast cell stabiliser ketotifen decreases visceral hypersensitivity and improves intestinal symptoms in patients with irritable bowel syndrome	41	2010
	8	Intestinal microbiota in functional bowel disorders: a Rome foundation report	37	2013
	9	Postinfectious irritable bowel syndrome	32	2009
	10	Rifaximin therapy for patients with irritable bowel syndrome without constipation	30	2011

### 4.4 Analysis of keywords, clusters, and burst terms

The research theme and scope of this field of study can be immediately understood through the keyword co-occurrence. The top 20 keywords in terms of frequency and centrality for IBS and visceral hypersensitivity from 2012 to 2022 are displayed in Table 4. What can we see from the table is that “irritable bowel syndrome” is the keyword with the highest statistical frequency, followed by “visceral hypersensitivity” and “mast cell”. What’s more, keywords such as “pain,” “abdominal pain,” “double-blind,” “activation,” “expression,” and “quality of life” were used more than 100 times, which reveals the current topic of research this field. Moreover, the gut microbiota requires attention in the first 20 keywords, outside of the pathogenesis keywords. Centrality represents the correlation between keywords. As shown in Table 4, the centrality of “colonic motility” ranks first, followed by “corticotropin-releasing factor,” “enteric nervous system,” “sensitivity” and “maternal separation.” Notably, these keywords combined IBS with visceral hypersensitivity and related other studies.

**TABLE 4:** Top 20 keywords in terms of frequency and centrality.

Ranking	Keyword	Frequency	Keyword	Centrality
1	IBS	744	colonic motility	0.1
2	visceral hypersensitivity	512	corticotropin releasing factor	0.08
3	mast cell	143	enteric nervous system	0.08
4	pain	140	sensitivity	0.07
5	abdominal pain	134	maternal separation	0.07
6	double blind	107	coliti	0.07
7	activation	101	hyperalgesia	0.06
8	expression	101	controlled trial	0.06
9	quality of life	100	chain fatty acid	0.05
10	symptom	97	gastrointestinal tract	0.05
11	visceral pain	95	neuron	0.05
12	functional gastrointestinal disorder	87	nerve growth factor	0.05
13	inflammation	75	modulation	0.05
14	colorectal distension	72	quality of life	0.04
15	gut microbiota	71	colorectal distension	0.04
16	mechanism	70	mechanism	0.04
17	model	68	ibs	0.04
18	hypersensitivity	68	colon	0.04
19	stress	65	*in vitro*	0.04
20	receptor	61	fecal microbiota	0.04

In order to understand the latest progress in studies with IBS and visceral hypersensitivity since 2012, we used CiteSpace to cluster the keywords of IBS and visceral hypersensitivity. Seven clusters were obtained, which are shown in Table 5. Generally speaking, the clustering effect is reasonable when the Sihouette is larger than 0.5 (Zou & Sun, 2021). The #0 cluster’ label is “probiotics”, followed by cluster #1 “visceral pain” and cluster #2 “mast cell”, representing the clusters newly developed since 2012.

**TABLE 5:** Keyword cluster analysis.

Cluster	Size	Sihouette	Mean year	Label (LLR)	Other keywords
#0	81	0.645	2014	probiotics	visceral hypersensitivity;placebo controlled trial; diet; small intestinal bacterial overgrowth
#1	76	0.578	2015	visceral pain	dorsal root ganglion; anterior cingulate cortex; histone acetylation; neonatal maternal deprivation
#2	70	0.496	2015	mast cell	visceral hypersensitivity; mast cells; enterochromaffin cell; glucagon-like peptide-1
#3	69	0.574	2015	abdominal pain	colonic distension; visceral sensation; hpa axis; spinal cord
#4	61	0.663	2015	prevalence	symptom; IBS; depression; risk factor
#5	23	0.889	2016	functional dyspepsia	gastric accommodation; overlap; postprandial distress syndrome; gastroesophageal reflux disease
#6	21	0.761	2017	serotonin	ankyrin 1; pi-ibs; pellino-1; inflammatory mediators
#7	20	0.844	2013	gastrointestinal tract	sensory neuron; transient receptor potential vanilloid 1; guanylate cyclase c; calcium channel

**FIGURE 7:**
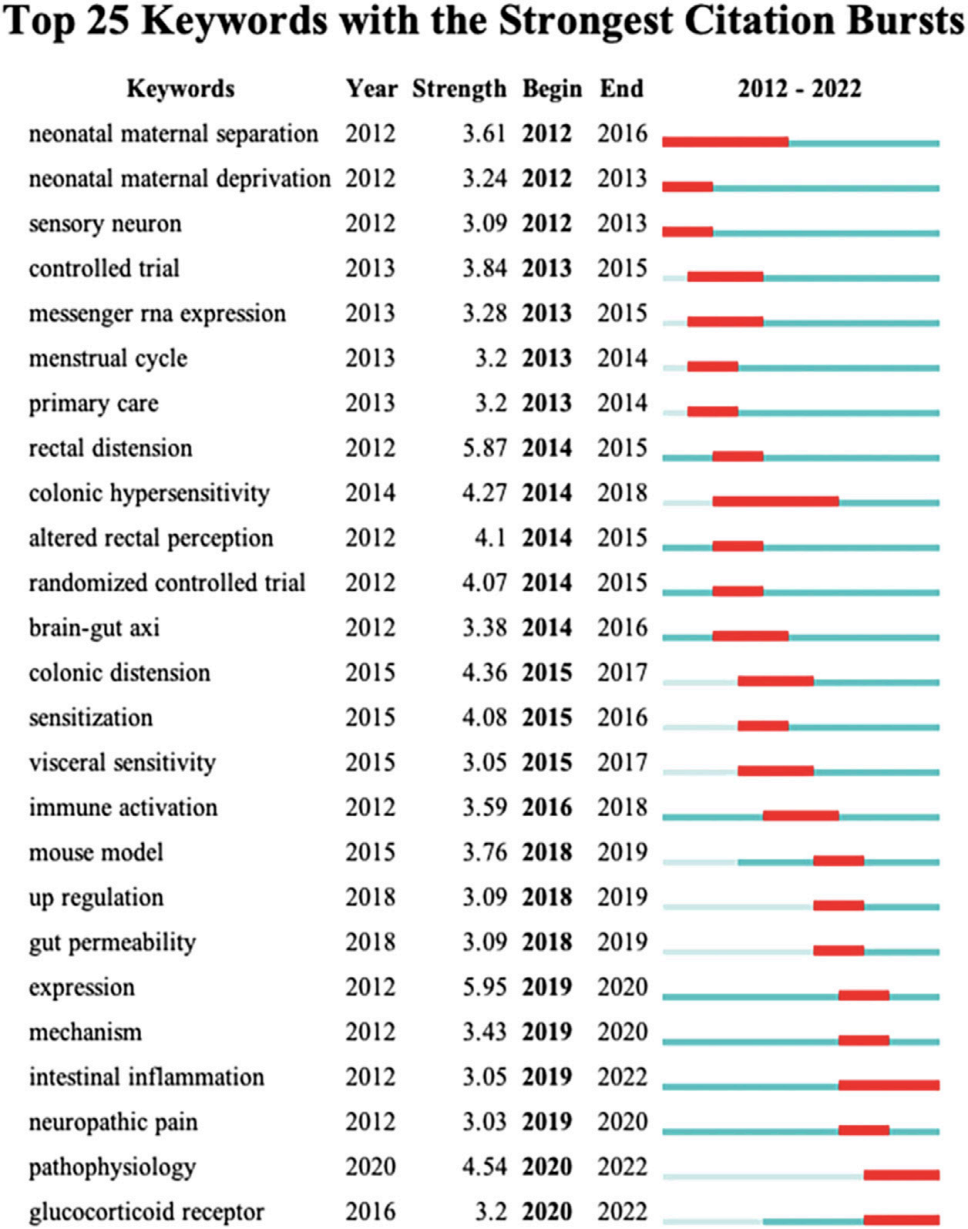
Top 25 keywords with the strongest citation bursts.

Similarly, keyword bursts describe a sudden surge in the research field over a period of time, which can indicate a prospective development trend for the research field. The top 25 terms in this research topic with the strongest burst intensity are shown in Figure 7. The length of the keyword bursts is shown by the red line. The keyword themes gradually changed from “rectal distension,” “colonic hypersensitivity,” “altered rectal perception,” and “randomized controlled trial,” as shown in the figure, to the current “mechanism,” “intestinal inflammation,” “neuropathic pain,” “pathophysiology,” and “glucocorticoid receptor” changes. This suggests that the physiological and pathological mechanism is the main focus of contemporary research on IBS and visceral hypersensitivity.

## 5 Discussion

### 5.1 The analysis of the general information

To identify research hotspots and frontiers, this study gathered data from the WoSCC over the previous 10 years. The annual number of publications generally displayed a steadily increasing trend. With 329 publications, China has the most publications published, followed by the US and Belgium. This indicates that China, as a developing country, has made considerable contributions and plays a more prominent role in the domains of IBS and visceral hypersensitivity research. At the same time, the United States is a major research power in the field, thanks to its strong economic power and scientific support.

In specific areas of research, bibliometric analysis can be used to assess collaboration among authors, institutions, and countries (Ma et al., 2021). The degree of closeness in cooperation is expressed by centrality. The stronger the cooperation, the greater the centrality. The top five countries in terms of central value are the United States, Germany, Australia, England, and China, indicating that these countries cooperate closely with other countries. Despite having the highest annual publication volume, China is less cooperative with other countries than the US. Cooperation among institutions reveals that China Acad Chinese Med Sci, Univ Hosp Leuven, Katholieke Univ Leuven, and Univ Michigan collaborate the most closely and have the highest centrality. This study suggests that China and the United States have a significant impact on the research of IBS and visceral hypersensitivity. Simren, Magnus, De Man, and Joris G worked closely with other researchers on author collaboration. The cooperation of other authors was zero, indicating that author cooperation needed to be strengthened. These authors’ collaboration may be related to the research field and financial support. There is a reason to believe that close collaboration among countries, institutions, and authors can help the long-term progress in this field.

### 5.2 Research focus and hot spot

Bibliometric analysis can also help scholars grasp the research hotspot and development trends in the research field. Based on highly co-cited references, highly cited references, and keyword clusters analysis, we found that the hot spots and trends of IBS and visceral hypersensitivity were closely related to their mechanisms, including mast cells, intestinal mucosal barrier, protease activity, TRPV1, etc., and the gut microbiota should be focused on. In addition, according to the keyword burst analysis, it can be seen that scholars have gradually deepened their understanding of IBS and visceral hypersensitivity, and entered into the research level of cellular and molecular mechanisms. Also, researchers can access the literature and research advancements connected to IBS and visceral hypersensitivity by selecting the most frequently used terms during the previous 10 years. What’s more, studies on probiotics and visceral hypersensitivity are also worthy of attention, which may contribute to the treatment of IBS.

### 5.3 Mechanisms of IBS and visceral hypersensitivity

Irritable bowel syndrome (IBS) is a functional bowel disorder, where symptoms are characterized by abdominal pain or discomfort, and changes in bowel habits (Borghini et al., 2017). Although the exact cause of IBS is unknown, changes in gastrointestinal motility, visceral hypersensitivity, postinfection reactivity, and gut-brain axis are all associated with the pathogenesis of IBS (Saha, 2014). As a result, more research into the mechanism of visceral hypersensitivity may help to reveal the mystery of IBS.

Mast cells could play a role in the pathogenesis of visceral hypersensitivity (Li et al., 2020). Studies have shown that mast cells cause changes in gastrointestinal function, and increasing mast cell numbers affect intestinal mucosal barrier permeability, which leads to the development of visceral sensitivity (Walker et al., 2011; Bednarska et al., 2017). Mast cells that surround nerve fibers normally act on them by releasing mediators like nerve growth factor (NGF), histamine, and trypsin (Grundy, 2004). Relevant studies have demonstrated that the levels of histamine and trypsin in colonic biological samples are related to the degree of visceral pain hypersensitivity in IBS mice (Buhner et al., 2009). Furthermore, psychological factors such as fatigue and depression are linked to an increase in intestinal mast cell count, implying that psychological factors may play a role in the development of IBS (Walker et al., 2011).

New studies have demonstrated the important role of intestinal permeability and immune activation in the development of IBS symptoms (Marshall et al., 2004; Shulman et al., 2014). Protease-activated receptor-2 (PAR-2) is a G-protein-coupled receptor that is expressed in epithelial cells, immune cells, and terminal afferents of the gastrointestinal tract (Jacob et al., 2005). Activated PAR-2 is closely associated with the development of pain and inflammation (Bao et al., 2014). Recent studies have shown that patients with IBS have increased levels of proteases in the intestinal luminal contents and intestinal mucosa, which activate downstream PAR-2 (Lee et al., 2010; Rolland-Fourcade et al., 2017). PAR-2 activation can increase the release of painful neuropeptides and cell permeability in the gut of patients with irritable bowel syndrome, leading to the occurrence of visceral hypersensitivity (Gecse et al., 2008; Liang et al., 2016).

TRP vanilloid 1 (TRPV1), a receptor that responds to acidosis, capsaicin, endogenous vanilloid, and heat, is one of the most studied receptors involved in the development of visceral hypersensitivity (Julius D, 2013). Transient receptor potential (TRP) cation channels exist in peripheral nerve endings, and they can be activated directly or indirectly by proinflammatory mediators, which play a crucial role in visceral nociception (Perna et al., 2021). TRP channels activate and signal to the central nervous system leading to visceral pain perception (Hughes et al., 2013). Changes in this process lead to the occurrence of visceral hypersensitivity (Balemans et al., 2017). Existing studies have shown that TRPV1 expression is increased both in preclinical models of visceral hypersensitivity and in rectal biopsies from IBS patients (Julius D, 2013). The upregulation and/or sensitization of TRP channels is now recognized as an important mechanism of visceral hypersensitivity (Wouters et al., 2016).

### 5.4 The visceral hypersensitivity and gut microbiota

Gut microbiota is a vital cause of visceral hypersensitivity (Li et al., 2020). Recent studies have found that microbes can regulate visceral hypersensitivity and the perception of pain (Pusceddu & Gareau, 2018). A correlation between visceral pain disorders such as IBS and microbial dysregulation has been demonstrated in patients (Dupont, 2014; Distrutti et al., 2016). Animal experiments and human studies suggest that gut microbiota may be an important mediator of the gut-brain axis (Borre et al., 2014). The regulation of gastrointestinal homeostasis is influenced by this two-way communication between the gut microbiome and the brain, which also has an impact on the function of the central nervous system, including mood, cognition, and visceral pain (Cryan et al., 2019). Fecal transplantation has been observed to improve symptoms in hospitalized patients with visceral pain (Pinn et al., 2014; Johnsen et al., 2018). As a result, the role of the gut microbiota may become a new target for future visceral pain treatments. Nonetheless, our understanding of the mechanisms by which the microbiome reduces visceral pain and the interaction between the gut and the brain is still in its infancy. The role of the microbiome in visceral pain has yet to be experimentally proven, and whether fecal transplantation can improve visceral pain by improving the microbiome has yet to be clinically proven.

### 5.5 The visceral hypersensitivity and probiotics

Probiotics can improve intestinal barrier function and immunity by regulating visceral sensitivity (Theodorou et al., 2014). Several studies in various animal models have shown that probiotics can reduce visceral hypersensitivity and visceral pain (Darbaky et al., 2017; Liu et al., 2020; McVey Neufeld et al., 2020; Arslanova et al., 2021). The experiment results show that the probiotic VSL#3 can improve the expression of gene subsets involved in colonic pain and inflammation, as well as reduce visceral pain in IBS rat models (Distrutti et al., 2013). Probiotics (such as *Bifidobacterium* or *Lactobacillus*) have been shown in animal models of IBS to reduce visceral pain by regulating nerve function (Kamiya et al., 2006; Ma et al., 2009) and normalize hypothalamic-pituitary-adrenal axis (HPA) function (Gareau et al., 2007). According to some research, probiotics may mediate visceral sensitivity through immune regulation and the role in enhancing the epithelial barrier (Gareau et al., 2010; Pagnini et al., 2010). There is also growing evidence that probiotics may reduce the risk of visceral sensitivity in animal models by modulating neural function (Desbonnet et al., 2010; McKernan et al., 2010). Although numerous studies have shown that probiotics are beneficial to the improvement of symptoms of IBS, the mechanism of probiotics and visceral hypersensitivity has not been fully understood and elucidation (Ait-Belgnaoui et al., 2018). Because of the low risk of probiotic treatment, this opens up a new avenue for treating visceral hypersensitivity and visceral pain.

## 6 Strengths and limitations

We can gain a better understanding of the key points, hot spots, and research frontiers in the current research field by using Citespace and Vosviewer for literature visualization analysis, and we can point out the research direction for researchers in this research field. Our study, however, has some limitations. To begin with, we only searched the WOSCC database and not all publications related to this study, which may have resulted in the omission of some influential literature. Second, this study did not ensure that every document was completely consistent with the search requirements. Finally, the quality of search articles varies greatly, which may have an impact on the accuracy of the results.

## 7 Conclusion

This study evaluated and visualized 974 Web of Science publications linked to IBS and visceral hypersensitivity from 2012 to 2022 using bibliometrics and visualization analysis. IBS and visceral hypersensitivity were shown to be steadily rising in terms of annual publications analysis. The countries with the most publications are China. Univ Oklahoma and Simren, Magnus are the most published institution and author in this research field. Furthermore, this study outlines the present research hotpots in this field, points out new research directions, and offers a thorough analysis of developments in IBS and visceral hypersensitivity. Through the analysis of keywords and references, it can be found that the physiological and pathological mechanism of IBS and visceral hypersensitivity is still the focus of this research field in recent years (including mast cells, protease activity, TRPV1, etc.), especially the research related to the mast cell. In addition, this study found that the mechanism of visceral hypersensitivity may be related to gut microbiota. Meanwhile, the relationship between probiotics and visceral hypersensitivity may also provide new therapeutic ideas for reducing visceral pain and the occurrence of IBS. This article is timely to promote future research in this field by analyzing the publications between IBS and visceral hypersensitivity.

## Data Availability

The original contributions presented in the study are included in the article further inquiries can be directed to the corresponding author.
